# Brain Protection by Methylene Blue and Its Derivative, Azur B, via Activation of the Nrf2/ARE Pathway in Cisplatin-Induced Cognitive Impairment

**DOI:** 10.3390/ph15070815

**Published:** 2022-06-30

**Authors:** Ekaterina P. Krutskikh, Daria V. Potanina, Natalia A. Samoylova, Mariya V. Gryaznova, Irina S. Sadovnikova, Artem P. Gureev, Vasily N. Popov

**Affiliations:** 1 Department of Genetics, Cytology and Bioengineering, Voronezh State University, 394018 Voronezh, Russia; krutskikh_ep@bio.vsu.ru (E.P.K.); potaninadaa@gmail.com (D.V.P.); nataliya.samoylova.2000@mail.ru (N.A.S.); mariya-vg@mail.ru (M.V.G.); ira-ivankina@yandex.ru (I.S.S.); pvn@bio.vsu.ru (V.N.P.); 2 Laboratory of Metagenomics and Food Biotechnology, Voronezh State University of Engineering Technology, 394036 Voronezh, Russia

**Keywords:** cisplatin, methylene blue, azur B, cognitive impairment, mitophagy, oxidative stress, gene expression, Nrf2/ARE

## Abstract

Cisplatin is a cytotoxic chemotherapeutic drug that leads to DNA damage and is used in the treatment of various types of tumors. However, cisplatin has several serious adverse effects, such as deterioration in cognitive ability. The aim of our work was to study neuroprotectors capable of preventing cisplatin-induced neurotoxicity. Methylene blue (MB) and AzurB (AzB) are able to neutralize the neurotoxicity caused by cisplatin by protecting nerve cells as a result of the activation of the Ntf2 signaling pathway. We have shown that cisplatin impairs learning in the Morris water maze. This is due to an increase in the amount of mtDNA damage, a decrease in the expression of most antioxidant genes, the main determinant of the induction of which is the Nrf2/ARE signaling pathway, and genes involved in mitophagy regulation in the cortex. The expression of genes involved in long-term potentiation was suppressed in the hippocampus of cisplatin-injected mice. MB in most cases prevented cisplatin-induced impairment of learning and decrease of gene expression in the cortex. AzB prevented the cisplatin-induced decrease of genes in the hippocampus. Also, cisplatin induced disbalance in the gut microbiome, decreased levels of *Actinotalea* and *Prevotella*, and increased levels of *Streptococcus* and *Veillonella*. MB and AzB also prevented cisplatin-induced changes in the bacterial composition of the gut microbiome.

## 1. Introduction

In the treatment of oncological diseases, drugs that have a cytotoxic effect are used [1], since they are capable of causing the death of tumor cells by intoxicating them and destroying intracellular structures [2]. One such drug that is widely used in clinical practice is cisplatin [3].

The biological action of cisplatin is manifested by direct covalent binding of DNA with the formation of intrastrand and interstrand crosslinks, which leads to a stop in DNA replication and cell division, followed by apoptosis [4,5,6]. Mitochondria are actively involved in many mechanisms of programmed cell death, damage to which not only leads to energy deficiency but can also induce cell death [7], therefore, for normal functioning, the cell must remove non-functional or damaged mitochondria. Cisplatin, in turn, can cause the mitochondrial release of cytochrome C [8], leading to mitophagy [9].

In addition to high oncological activity, cisplatin also has very strong toxic properties that affect many organs [10]. For example, this drug induces pathogenesis in the cells of the nervous system [11,12]. Neurotoxicity of cisplatin is associated with its action on DNA, since cisplatin leads to molecular cross-linking of DNA in the cells of the nervous system, as well as its ability to cause chronic inflammation in the brain [4,5]. Therefore, one of the serious side effects associated with cancer treatment is the deterioration of cognitive abilities in patients who have undergone chemotherapy [13]. Due to this, some researchers use cisplatin as a chemical model for neurotoxicity [14,15,16,17].

Another serious side effect of cisplatin is an effect on the bacterial composition of the gut microbiome. Cisplatin administration caused gut microbiota dysbiosis in mice [18], led to the increased proportion of Bacteroidetes and reduced Firmicutes compared to controls, and decreased Shannon alpha diversity [19]. Gastrointestinal mucositis also is a cisplatin-induced serious side effect [20]. In recent years, accumulating data confirm that bacterial composition gut microbiomes may modulate cognitive function via a microbiome-gut–brain axis [21]. Cisplatin-induced dysbiosis leads to epithelial sloughing and mucosal ulceration of villous tips [22]. Epithelial sloughing causes the depletion of the gut barrier and may mediate chronic inflammation [23]. The inflammation may eventually spread from peripheral tissue to the brain and can cause neuroinflammation, which is an important causal mechanism in cognitive decline [24].

**Figure 1:**
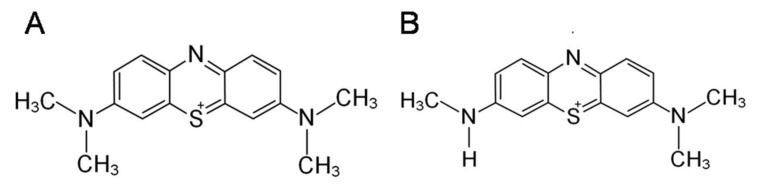
Chemical formulas of neuroprotective compounds: (**A**) methylene blue, (**B**) Azur B.

MB, (Figure 1A), due to its antioxidant properties, can potentially act as a neuroprotective substance, protecting cells of the nervous system from the effects of oxygen metabolites that induce the development of oxidative stress, thereby being a promising drug in the treatment of neurodegenerative diseases. It can transfer electrons in the electron transport chain from NADH and FADH2 directly to cytochrome C, bypassing complexes I–III; thereby improving mitochondrial function with affected complexes [25]. It is known that a low concentration can modulate the composition of the gut microbiome [26,27]. Correlation analysis showed that MB-induced changes in the bacterial composition of the gut microbiome correlate with the improved cognitive phenotype of mice. Also, among the thiosine dyes with neuroprotective properties, AzB (Figure 1B) can be distinguished, which in its structure and mode of action is similar to MB, however, has been studied to a lesser extent.

In this work, we explore the possibility of using MB and its structural analogue, AzB, as neuroprotective substances that can potentially reduce the negative effect of cisplatin on the cortex and hippocampus of the brain, as the main brain compartments responsible for memory formation [28]. The effectiveness of the pharmacological intervention on the cognitive functions of mice with cisplatin-induced memory impairment was evaluated in conjunction with the study of mitochondrial functions of the brain and microbiome–gut–brain axis.

## 2. Results

### 2.1. Open Field

When conducting the physiological test “Open Field”, eight different parameters were examined (hole-poking, number of grooming acts, length of grooming time, number of visits to the center, total time spent in the center, number of defecation acts, vertical activity, and horizontal activity). Statistically significant differences were found only in the duration of grooming of mice, between groups treated with thiosine dyes (MB and AzB) (U test *p* = 0.0086). Mice given AzB spent 80% less time grooming compared to mice given MB (Table 1).

### 2.2. Morris Water Maze

#### 2.2.1. Distance

**Figure 2:**
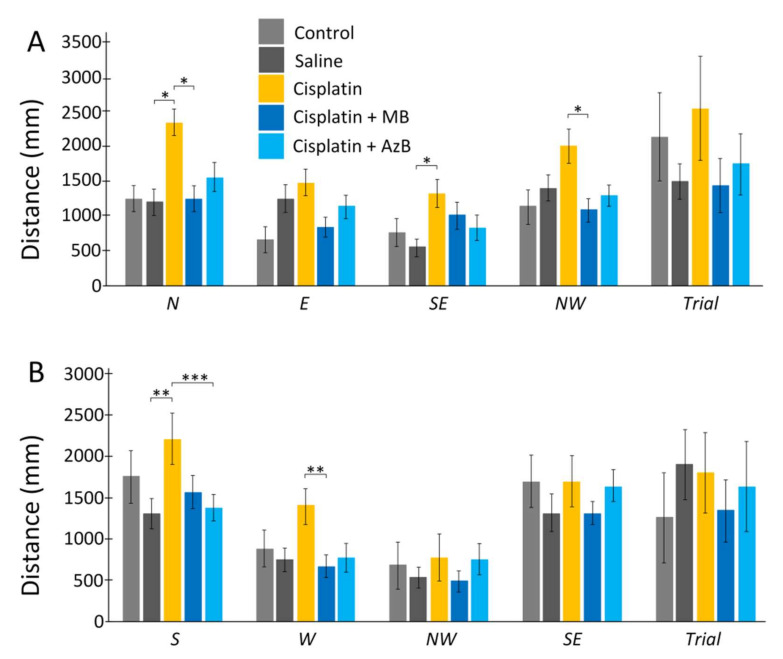
The Morris water maze. Distance in the (**A**) acquisition, (**B**) reversal learning. The results expressed as means ± SEM. Control *n* = 4, Saline = 7, Cisplatin = 6, Cisplatin+ MB = 8, Cisplatin+ AzB = 8. * *p* < 0.05, ** *p* < 0.01, *** *p* < 0.001, groups were compared using the U test.

Cisplatin injections and thiazine dyes did not affect the distance that mice swam on the 6th day of the experiment and the 12th day of the experiment. During direct training swimming, statistically, significant differences were found for the N quadrant. Mice injected with cisplatin swam 49% more distance compared to mice injected with saline (U test *p* = 0.036). Differences were also found between mice injected with cisplatin and mice treated with MB. Cisplatin-treated mice swam more by 47% (U test *p* = 0.034). Differences were when starting from the SE quadrant, and mice injected with cisplatin swam 59% more than mice injected with saline (U test *p* = 0.011). Differences were shown for mice treated with MB and mice treated with cisplatin injections alone when starting with NW. Cisplatin mice swam a greater distance in search of a platform by 46% (U test *p* = 0.02) (Figure 2).

During reversal training, the differences were when starting from the S quadrant. Mice given only cisplatin injections swam a greater distance compared to mice given saline injections by 41% (U test *p* = 0.0049) and mice given AzB by 38% (U test *p* = 0.001). When run from the W platform, statistically significant results were found between mice injected with cisplatin and mice treated with MB. Mice that consumed MB spent 51% less distance searching for the platform (U test *p* = 0.0073) (Figure 2).

#### 2.2.2. Time

**Figure 3:**
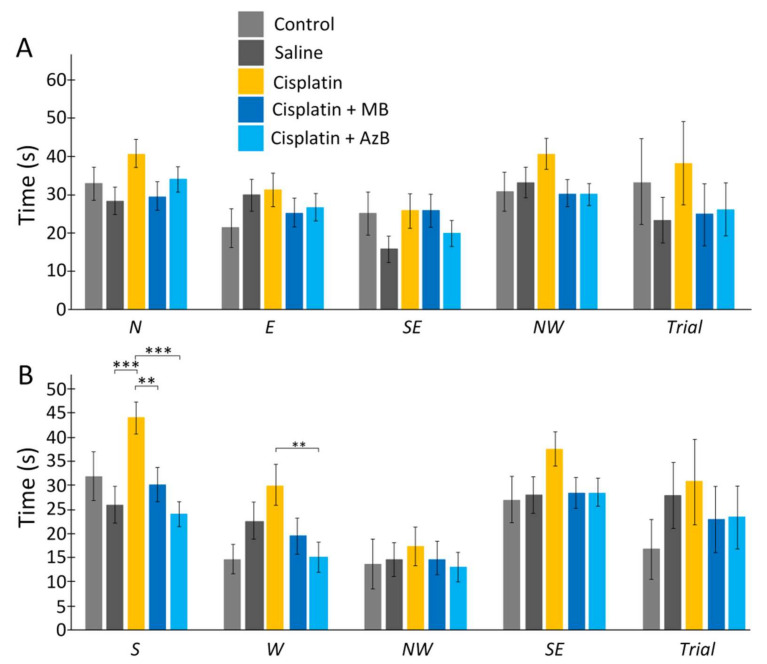
The Morris water maze. Time in the (**A**) acquisition, (**B**) reversal learning. The results expressed as means ± SEM. Control *n* = 4, Saline = 7, Cisplatin= 6, Cisplatin+ MB = 8, Cisplatin+ AzB = 8. ** *p* < 0.01, *** *p* < 0.001, groups were compared using the U test.

Cisplatin injections and thiazine dyes did not affect the time the mice spent searching for the platform on day 6 of the experiment and day 12 of the experiment. Mice that received cisplatin injections spent more time learning to swim than mice that received saline injections. This pattern was observed when starting from each quadrant (44% more when starting from N, 5% more when starting from E, 65% more when starting from SE, 23% more when starting from NW, 69% more when run with S (U test *p* = 0.00043), 34% more when run with W, 18% more when run with NW, 34% more when running with SE. However, U test showed no statistically significant difference for all quadrants except S (Figure 3).

The use of thiazine drugs affected the time during learning, usually during reverse learning. There were differences when starting mice from the S quadrant. MB-treated mice spent 30% less time than mice treated with cisplatin injections alone (U test *p* = 0.0053) and mice treated with AzB spent 44% less time than mice treated with cisplatin injections alone (U test *p* = 0.00001). Differences were found when starting mice from the W quadrant. Mice treated with AzB spent half as much time searching for the platform as mice treated with cisplatin injections only (U test *p* = 0.0074) (Figure 3).

### 2.3. Gene Expression Level

**Figure 4:**
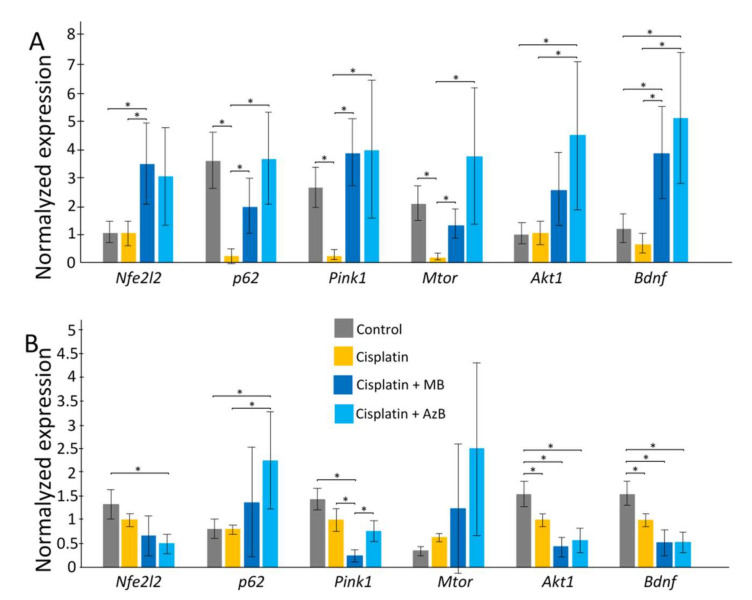
Expression of transcriptional factors in the (**A**) cortex, (**B**) hippocampus. The results expressed as means ± SEM. Control *n* = 11, Cisplatin = 6, Cisplatin + MB = 8, Cisplatin + AzB = 8. * *p* < 0.05, comparison of control group and treated groups using Tukey’s post-hoc test.

**Figure 5:**
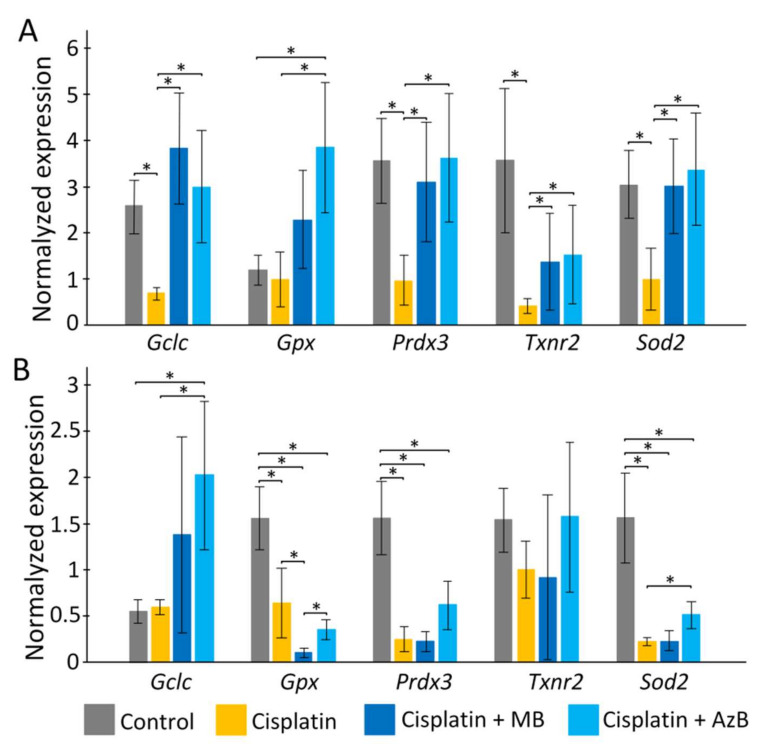
Expression of antioxidants in the (**A**) cortex, (**B**) hippocampus. The results expressed as means ± SEM. Control *n* = 11, Cisplatin= 6, Cisplatin + MB = 8, Cisplatin + AzB = 8. * *p* < 0.05, comparison of the control group and treated groups using Tukey’s post-hoc test.

In the cortex, cisplatin injections cause a sharp decrease in the expression of *p62*, *Pink1*, *Mtor* (all *p* < 0.05). The use of MB and AzB prevented the decrease in the expression of these genes in the cortex. In addition, MB caused an increase in *Nfe2l2* and *Bdnf* gene expression relative to control and cisplatin-injected mice (all *p* < 0.05). AzB induced an increase in *Akt1* and *Bdnf* gene expression relative to control and cisplatin-injected mice (all *p* < 0.05) (Figure 4). In mice injected with cisplatin, there was a decrease in the expression of several antioxidant genes such as *Gclc*, *Prdx3*, *Txnr2*, *Sod2* (all *p* < 0.05) in the cortex. MB and AzB prevented the cisplatin-induced decrease in the expression of *Gclc*, *Prdx3*, *Sod2* in the cortex (all *p* < 0.05). AzB induced an increase in *Gpx* expression relative to control and cisplatin-injected mice (all *p* < 0.05) (Figure 5).

In the hippocampus, cisplatin caused a decrease in the expression of *Akt1*, *Bdnf* (all *p* < 0.05). AzB increased *p62* gene expression relative to control and cisplatin-injected mice (all *p* < 0.05) (Figure 4). At the same time, AzB induced a decrease in the expression of the *Nfe2l2*, *Akt1*, *Bdnf* genes relative to the control in the hippocampus (all *p* < 0.05). MB induced a decrease in the expression of *Pink1*, *Akt1*, *Bdnf* genes relative to the control in the hippocampus (all *p* < 0.05) (Figure 4). Injections of cisplatin into the hippocampus induced a decrease in the expression of *Gpx*, *Prdx3*, *Sod2* (all *p* < 0.05). AzB therapy prevented the cisplatin-induced decrease in *Sod2* expression (*p* < 0.05) and also increased *Gclc* expression (*p* < 0.05). MB did not interfere with the cisplatin-induced decrease in antioxidant gene expression; on the contrary, it reduced *Gpx* expression relative to control and cisplatin-injected mice (all *p* < 0.05) (Figure 5).

### 2.4. The Amount of mtDNA Damage

**Figure 6:**
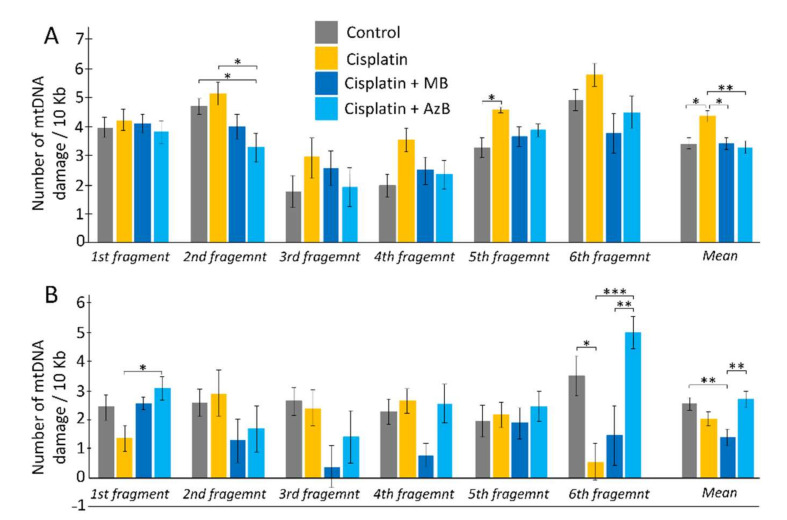
Copy number of mtDNA in the (**A**) cortex, (**B**) hippocampus. The results expressed as means ± SEM. Control *n* = 11, Cisplatin = 6, Cisplatin + MB = 8, Cisplatin + AzB = 8. * *p* < 0.05, ** *p* < 0.01, *** *p* < 0.001 comparison of the control group and treated groups using Tukey’s post-hoc test.

When performing calculations to study damage to the mtDNA cortex, statistically significant differences were found for the 2nd long fragments (F(3,108) = 3.846, *p* = 0.012) between mice that consumed AzB and the control group (30% more in control mice; post hoc Tukey test *p* = 0.031) and mice injected with cisplatin (37% more in mice injected with cisplatin; post hoc Tukey test *p* = 0.023). Differences were observed for the 8th long fragment (F(3, 108) = 2.4492, *p* = 0.068) between control and mice injected with cisplatin (28% greater in mice injected with cisplatin; post hoc Tukey test *p* = 0.047). For the 9th long fragments (F(3, 108) = 2.039, *p* = 0.113), differences were between mice injected with cisplatin and mice treated with MB (35% greater in mice injected with cisplatin; post hoc Tukey test *p* = 0.094). When summing mtDNA damage for all long fragments (F(3, 668) = 4.089, *p* = 0.007), significant differences were found between the control group of mice and mice injected with cisplatin (21% more in mice injected with cisplatin; post hoc Tukey test *p* = 0.014); for mice treated with MB and mice treated with injections of cisplatin (21% more in mice treated with injections of cisplatin; post hoc Tukey test *p* = 0.023); for mice treated with AzB and the group receiving cisplatin injections (25% more for mice treated with cisplatin injections; post hoc Tukey test *p* = 0.006) (Figure 6).

When calculating the amount of hippocampal mtDNA damage, differences were found for the 1st long fragment (F(3, 48) = 3.152, *p* = 0.033) between mice injected with cisplatin and mice that consumed AzB (55% more in mice fed AzB; Tukey’s post hoc test *p* = 0.022). For the 3rd long fragment (F(3, 48) = 2.319, *p* = 0.087) differences were observed between the control group and the MB group (85% more in the control group; post hoc Tukey test *p* = 0.088). Differences were found for long fragment 7 (F(3, 48) = 3.023, *p* = 0.039) between mice treated with MB and mice treated with cisplatin injections (70.5% more in mice treated with cisplatin injections; post hoc Tukey test *p* = 0.055), as well as between mice that received AzB (69.5% more in mice fed AzB; post hoc Tukey test *p* = 0.073). For 9 long fragments (F(3, 48) = 7.504, *p* = 0.0003), differences were between cisplatin-injected and control mice (84% greater in control mice; post hoc Tukey test *p* = 0.016), as well as mice treated with AzB (88% more in mice treated with AzB; post hoc Tukey test *p* = 0.0006). Differences were between mice treated with MB and AzB (71% greater for AzB; Tukey’s post hoc test = 0.009). When mtDNA damage was summed for all long fragments (F(3, 308) = 5.424, *p* = 0.001), significant differences were found between mice from the control group and mice treated with MB (46% more in control; post hoc Tukey’s test *p* = 0.004) and between mice treated with thiosine dyes (48% more in mice treated with AzB; post hoc Tukey’s test *p* = 0.0025) (Figure 6).

### 2.5. Bacterial Composition of the Gut Microbiome

**Figure 7:**
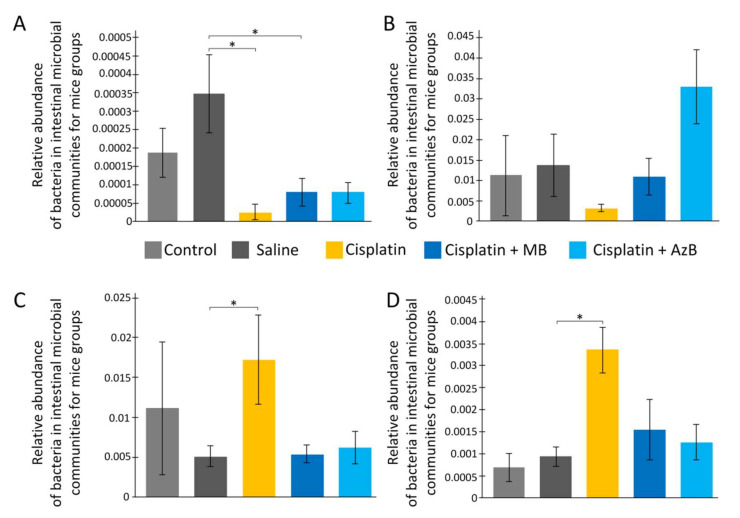
The content of predominant bacteria in the gut microbiome. (**A**) *Actinotalea*, (**B**) *Prevotella*, (**C**) *Streptococcus*, (**D**) *Veillonella*. * *p* < 0.05 comparison of the control group and treated groups using Tukey’s post-hoc test.

Cisplatin intake affects the bacterial composition of the gut microbiome. As a result of the analysis, we found a change in the number of *Actinotalea* (class *Actinobacteria*) (F(4, 18) = 3.759, *p* = 0.022). Cisplatin injections reduce *Actinotalea* levels 15-fold (post hoc Tukey’s test, *p* < 0.05). MB and AzB pre-treatment did not prevent cisplatin-induced changes in the *Actinotalea* abundance. There is a change in the level of *Prevotella* (class *Bacteroidia*) (F(4, 18) = 2.599, *p* = 0.074). Cisplatin injections reduce *Prevotella* levels four-fold (post hoc Tukey’s test, *p* = 0.057). The level of *Prevotella* in the MB and AzB-treated group was similar to saline-injected mice (Figure 7).

There are changes in the level of *Streptococcus* (class *Bacilli*, phylum *Firmicutes*) (F(4, 18) = 3.119, *p* = 0.041). On the contrary, cisplatin injections lead to an increase of *Streptococcus* abundance 3.4-fold compared to saline-injected mice (post hoc Tukey’s test, *p* < 0.05). MB and AzB pre-treatment prevent the increase of *Streptococcus* level. Post hoc Tukey’s test showed *p* = 0.052 during comparison of MB-treated and cisplatin-injected-only mice, and *p* = 0.097 during comparison of AzB-treated and cisplatin-injected-only mice. Similar changes were noted for the *Veillonella* (class *Negativicutes*, phylum *Firmicutes*) (F(4, 18) = 3.937, *p* = 0.018). Cisplatin injections lead to an increase of *Veillonella* level 3.6-fold compared to saline-injected mice (post hoc Tukey’s test, *p* < 0.05). Only AzB pre-treatment prevented *Veillonella* level increasing (post hoc Tukey’s test, *p* = 0.054), not MB (post hoc Tukey’s test, *p* = 0.138) (Figure 7).

## 3. Discussion

LTM is a vast store of knowledge and a record of prior events [29]. LTM is supported by stronger and more stable neuronal connections compared with short-term memory and working memory [30]. The process of strengthening these connections that results in the LTM formation is long-term potentiation (LTP), which takes place mostly in the hippocampus [31]. LTP impairments are involved in various pathologies associated with the deterioration of cognitive functions, including dementia, neurodegenerative diseases, and age-related senility [32]. Several cytotoxic compounds including cisplatin may facilitate impairment of LTP. Cisplatin-induced cognitive impairment was documented in animals models using Morris water maze [33,34,35,36,37,38,39,40], Y-maze test [33,41,42,43,44], T-maze [45,46,47], and novel object recognition test [9,48,49,50,51], including in humans in the treatment of oncological diseases using chemotherapy using cisplatin as a chemostatic [12,52,53,54,55].

We have shown that cisplatin does not impair memory on trial days, but there was an increase in the duration of the platform search and the distance the mouse traveled in search of the platform on training days. That is, we can conclude that cisplatin still had a depressing effect on the LTM of mice. It can be assumed that this is due to the inhibition of signaling pathways that are involved in the formation of LTM. LTP is a complex process that requires the production of large amounts of proteins. mTORC1 (mammalian target of rapamycin complex 1) activation in dendrites promotes proteins synthesis, which is necessary for neurogenesis and LTP formation [56]. The mTORC1 is regulated by the PI3K/AKT signaling pathway. PI3K is a protein, which catalyzes phosphatidylinositol 4,5-bisphosphate (PIP2) conversion into phosphatidylinositol 3,4,5-trisphosphate (PIP3). This process is essential for the downstream phosphorylation of PDK1 (phosphoinositide-dependent kinase 1) and AKT (protein kinase B, PKB) [57]. In turn, the PI3K-mTORC1 signaling is activated via BDNF (brain-derived neurotrophic factor) binding [58]. We found that cisplatin causes a decrease in the expression of the *Mtor* gene in the cortex, and of the *Akt1* and *Bdnf* genes in the hippocampus. Thus, we may suggest that the cisplatin-induced decrease in LTP is associated with molecular mechanisms of signal transduction through the BDNF-PI3K-mTORC1 axis. Data from other studies demonstrate a decrease in *Bdnf* gene expression [59].

Mitochondrial dysfunction may be another reason for the cisplatin-induced decrease in LTP. We found that the amount of mtDNA damage in cisplatin-treated mice is increased in the cortex but not in the hippocampus. Also in the hippocampus, cisplatin significantly reduced the level of expression of genes that are responsible for mitophagy (*Pink1* and *p62*). Mitophagy is the selective degradation of mitochondria by autophagy. Mitophagy can promote the elimination of damaged or stressed mitochondria. This process is essential for cellular health [60]. Mitophagy dysregulation is one of the processes, which leads to memory dysfunction under various neurodegenerative pathologies and aging [61]. Cisplatin induces mitochondrial degradation and damages mitochondrial DNA [9].

Another link in the chain of mitochondrial dysfunctions may be disorders in the antioxidant defense of the brain. The expression of most antioxidant genes was strongly reduced in both brain regions studied in cisplatin-treated mice. In general, cisplatin reduces the level of expression of genes of the antioxidant system [62].

The introduction of cisplatin caused a state of microbial imbalance in the body. Cisplatin reduces the level of bacteria of the genus *Actinotalea*, which belong to the class *Actinobacteria*. These bacteria can produce acetate, which has great inhibitory activity against pathogenic species that cause infections caused by ingestion of substances harmful to the body [63]. They are associated with anti-inflammatory properties, reduce intestinal permeability, intestinal lipopolysaccharide levels, and improve intestinal mucosal barrier properties in mice [64]. Cisplatin also caused a reduction in the level of *Prevotella* (*Bacteroidia* class). These bacteria contribute to the improvement of glucose metabolism, which is stimulated by the intake of prebiotics, and *Prevotella* can have an immunomodulatory effect on the body [65,66]. In contrast, cisplatin increased the number of *Streptococcus* (class *Bacilli*). These bacteria are found in the normal intestinal microflora, however, with an increase in their level, a pathogenetic effect is observed associated with the production of toxins: hemolysin, streptolysin, streptokinase A and B, hyaluronidase, deoxyribonuclease [67]. The number of *Veillonella* (class *Negativicutes*) also increased. Bacteria of the genus *Veillonella* have been found in high numbers in the microbiome of organisms suffering from depression and therefore cognitive impairment [68].

Thus, the negative effect of cisplatin on the cognitive abilities of mice is complex. It is associated with neurogenesis dysfunction, mitochondrial dysfunctions, which are manifested in mtDNA damage, reduced expression of antioxidants and genes responsible for mitophagy, as well as disorders of intestinal microbiome homeostasis, which can lead to increased inflammatory processes. To prevent these dysfunctions, we used MB.

Although MB was used in medicine for over 120 years [69], the neuroprotective effect of MB began to be studied in the late 1970s when it was shown that MB produced a significant retrograde enhancement of learning [70]. Later, it has been shown that MB treatment during the memory-consolidation period restored the memory retention impaired by the inhibition of cytochrome oxidase [71]. MB improved cognitive function in 15-month-old mice in Morris water maze. In particular, MB increased the values of short-term memory and caused a decrease in the distance that mice spent searching for the platform during the learning phase for LTM estimation [27]. The randomized, double-blinded, placebo-controlled clinical trial showed that MB administration increased response in the bilateral insular cortex during a psychomotor vigilance task and was also associated with a 7% increase in correct responses during memory retrieval [72]. In this study we did not find statistically significant improvement of LTM values, although there is a tendency to decrease the time distance for the search of the platform both for acquisition and reversal trials. Statistically significant improvements to these values were obtained during the learning stage of the experiment, where there were more measurements compared with trials.

We can assume that the therapeutic effect of MB on LTM is associated with the activation of the Nrf2/ARE signaling pathway [32], because MB treatment led to an increase of *Nfe2l2* gene expression. There are several mechanisms that MB can lead to the activation of Nrf2. MB can accept electrons from NADH and perform alternative electron transport in the electron-transport chain [73]. This leads to a shift in the NAD^+^/NADH equilibrium toward NAD^+^. This leads to the activation of AMP-activated protein kinase (AMPK), which can activate the Nrf2 [74]. It is well-known that the Nrf2/ARE pathway is a major determinant of phase II gene induction, mainly antioxidant enzymes [75]. We showed that MB prevented the cisplatin-induced decrease in most antioxidant gene expression in the cortex. The effect of MB is not so clear-cut in the hippocampus.

There is crosstalk between Nrf2/ARE signalling and mitophagy crosstalk. The p62 protein is a selective mitophagy receptor for the degradation of ubiquitinated substrates [76]. There is evidence that p62 can positively regulate the Nrf2/ARE signal pathway by binding to KEAP1 (Nrf2 negative regulator). However, Nrf2 up-regulates *p62* expression. Thereby, a regulatory loop is formed between two important signaling pathways [76]. Also, Nrf2 regulates the expression of *Pink1*. PINK1 is necessary for activation of PARKIN, which can ubiquitinate several proteins on the outer mitochondrial membrane, which triggers mitophagy [77]. MB treatment increased expression of *p62* and *Pink1* in the cortex compared with cisplatin-treated mice, not in the hippocampus.

It is supposed that the Nrf2/ARE signal pathway may have a direct impact on synaptic plasticity [32]. Nrf2/ARE signaling is closely associated with the PI3K/AKT signaling pathway [78]. The promoter of the *Mtor* gene has an ARE sequence, therefore Nrf2 may have a direct impact on the *Mtor* expression [79]. *Bdnf* is also an Nrf2-targeted gene [80]. We observed MB-related prevention of cisplatin-induced decrease in *Mtor* and *Bdnf* expression in the cortex. In this regard, we can assume that the cognitive improvements that were caused by MB are also associated with the positive regulation of synaptic plasticity through the BDNF-PI3K-mTORC1 axis.

It is known that in organisms MB is metabolized to yield the N-demethylated metabolites, AzB as major metabolite and azure A as secondary metabolite [81]. While AzB is structurally similar to MB, it differs for the ionization state of the oxidized form [82]. For biological efficiency, it is probably of advantage that, in contrast to oxidized MB, oxidized AzB can assume a neutral quinoneimine form that readily diffuses through membranes [69]. There is evidence that AzB as well as MB suppresses the expression of *App* and *Bace1*, which are responsible for Tau aggregation and the development of Alzheimer’s disease [83] and inhibit caspases [84]. AzB is also effective in inhibiting the pro-inflammatory cytokine, tumor necrosis factor (TNFα, Tumor necrosis factor-alpha) [85]. It has been shown that AzB can be used as an antidote for cyanide poisoning, even at lower concentrations than MB. The concentration of AzB 4 mg/kg on the therapeutic effect corresponds to the concentration of 20 mg/kg MB [86]. AzB is a more potent reversible inhibitor of monoamine oxidase (MAO) than MB, which makes it a promising antidepressant and anxiolytic drug [82].

Our data indirectly confirm the anxiolytic properties of AzB. Cisplatin-treated mice that received AzB spent 80% less time grooming compared to mice that received MB (Table 1), since it is well known that mouse grooming is a reliable anxiety marker [87]. The AzB-related improvements in cognitive ability are broadly comparable to MB-related improvements. Although, AzB-treated mice had 22% more distance compared with MB-treated mice for reaching the platform during the acquisition trial and 22% more during the reversal trial. Differences are statistically insignificant. Only during starting from one position (S) did AzB-treated mice search the platform earlier than cisplatin-injected mice, while MB-treated mice searched the platform early while starting from three positions (N; NW; W). Simultaneously, AzB had only 5% more time to search the platform compared with MB-treated mice during the acquisition trial and 1,6% more during the reversal trial. On the contrary, AzB-treated mice found the platform faster than cisplatin-injected mice when starting from two positions (S; W), while for MB-treated mice it was only when starting from one position (S). So, these data may indicate the same protective effect of both substances on the cognitive functions during the cisplatin-induced disturbance. Effect of AzB on the gene expression in the cortex comparable with the effect of MB. In the hippocampus, AzB increased expression in the *p62* compared with control and cisplatin-injected mice and *Pink1* compared with MB-treated mice. In the hippocampus, AzB increased expression of *Gpx* and *Sod2*, unlike MB. This effect may be caused by structural features, which allow AzB to penetrate the blood–brain barrier better than MB [69].

**Figure 8:**
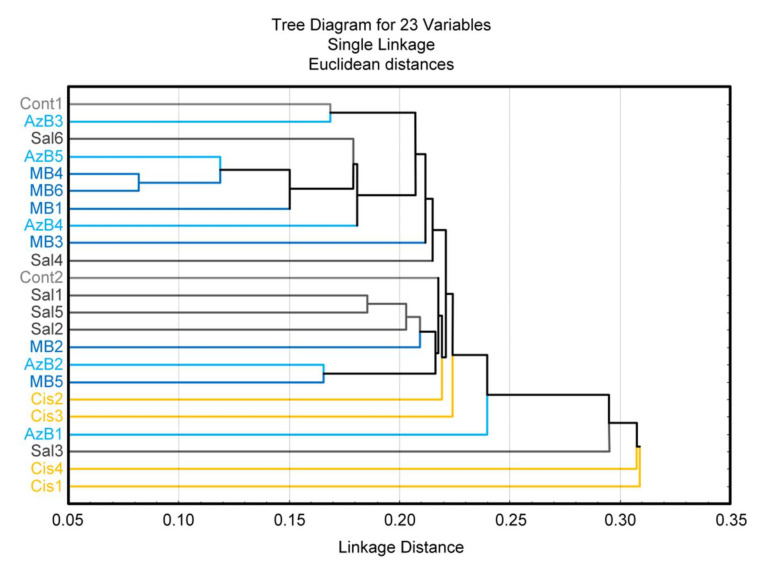
Cluster analysis.

Both AzB and MB affect the bacterial composition of the gut microbiome similarly. These compounds increased the level of *Prevotella* and decreased the level of *Streptococcus* and *Veillonella* compared with cisplatin-treated mice. To a certain extent, the AzB effect was more pronounced than the MB effect. Analysis of distance-based clustering based on mice microbial structure showed that the control group, saline-injected mice, most of MB and AzB-treated mice clustered in groups with low Euclidean distance, while all cisplatin-injected mice are outside these groups (as an exception, there isone saline-injected and one AzB-treated mouse in the outside group) (Figure 8). These data may suggest that cisplatin causes significant changes in the structure of the bacterial composition of the gut microbiome. These changes did not occur in the group of mice that received AzB and MB, so these mice clustered in groups with the control mice.

## 4. Materials and Methods

### 4.1. Animals

Three-month-old mice (C57Bl/6 strain) were used in the experiment. Mice were obtained from Stolbovaya Nursery (Settlement Stolbovaya; Moscow region, Russia) and kept under standard conditions of vivarium at t = 25 °C, relative humidity of at least 40%, and a 12-h light/dark cycle, mice had access to food and water without restrictions. Experimenter obtained randomly encoded mice for the blinded study of the conducting behavioral tests.

### 4.2. Design of Experiment

Mice were divided into five groups: the first group was the control (mice received only food and water), the second group was injected with saline at a dose of 2 mg/kg/day, the third group was exposed to cisplatin in the form of intraperitoneal injections at a dose of 2 mg /kg/day, the fourth group received injections of cisplatin (2 mg/kg/day) and oral MB at a dosage of 15 mg/kg/day, mice from group 5 received injections of cisplatin (2 mg/kg/day) and took AzB at a dosage of 15 mg/kg/day.

**Figure 9:**
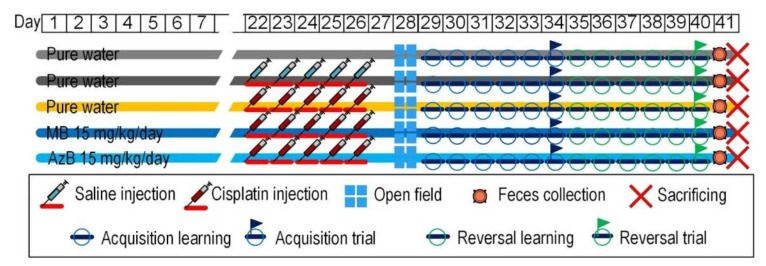
Design of experiment.

The experiment was carried out for three weeks. The mice received cisplatin injections during the first week, and the mice received thiosine dyes throughout the experiment. At the end of the administration of cisplatin injections to mice, physiological tests were carried out to assess the cognitive and behavioral functions of the animals, namely the Open Field test and the Morris water maze. After that, the mice were sacrificed by the rules established by the ethical commission of the Voronezh State University (Section of Animal Care and Use, 94 protocol 42-03 of 8 October 2020). The hippocampus and the cerebral cortex were removed from the experimental animals for the molecular part of the experiment, namely the isolation of nucleic acids (Figure 9).

### 4.3. The “Open Field” Test

The animal was placed in an open space (arena), where it was kept for 5 min. The arena is a square wooden box, the open field of which is marked with lines to determine the position of the animal. A new environment can elicit two types of responses: exploratory behavior aimed at familiarizing the animal with the environment, and defensive behavior when fear arises. In this experiment, we evaluated such indicators as the level of interest in “minks” (artificial holes at the bottom of the box), the number of grooming acts, the length of the grooming time, the number of exits to the center, the total time spent in the center, the number of defecation acts, vertical activity, and horizontal activity.

### 4.4. The “Morris Water Maze” Test

To assess the cognitive abilities of mice, we conducted a physiological test: “Morris Water Maze”. It consisted of the fact that we had to evaluate the ability of mice to navigate in space by remembering the location of a stationary object-platform, which was hidden under a layer of colored water so that the mice could not see it. To do this, we allotted five training days and one control day (for direct and reverse training). The mouse was launched from fixed points of the poolside into the water (during training, four attempts were given and one attempt on the control day of the launch), while the mouse had to orient itself and find the platform by climbing onto it. As a result, to observe the improvement or deterioration of the LTM (long-term memory) of mice, we used parameters such as time (the time the mouse spent searching for the platform) and distance (the distance the mouse swam). The difference between direct and reverse learning was that the platform in the process of reverse learning was located opposite to how it was exposed during direct learning (Table 2).

### 4.5. Gene Expression Level Assessment

Isolation of total RNA was carried out using the commercial ExtractRNA kit according to the attached protocol. Reverse transcription was performed using an “Eppendorf Mastercycler personal” instrument. Quantitative PCR analysis was performed on a “Bio-Rad CFX96TM Real-Time System, Hercules, CA, USA” instrument. The reaction mixture (volume 20 µL) included: Encyclo polymerase (0.4 µL), 10 × Encyclo Buffer (2 µL), dNTP (0.4 µL) (Evrogen, Moscow, Russia), 20× SYBR Green Master Mix (1 µL) (BioDye, Moscow, Russia), 1 µL of a mixture of forward and reverse primers.

General denaturation was carried out at 95 °C for 3 min; denaturation at the beginning of the cycle 95 °C 30 s; primer annealing 61 °C 30 s, elongation 72 °C 30 s; the number of cycles 38; total elongation 72° 5 s; melting curve from 65 °C to 95 °C. Quantitatively normalized gene expression was expressed in relative fluorescence units (RFU).

The primer sequences were as follows (Table 3):

### 4.6. Measurement of mtDNA Damage

The amount of mtDNA damage was estimated by quantitative long-range PCR. Each PCR reaction contained 1× of Encyclo polymerase, 1× Encyclo buffer, 0.2 mM of each dNTP (all Evrogen, Moscow, Russia), 1× SYBR GreenMasterMix (BioDye, Moscow, Russia), and a mix of forward and reverse primers in a total volume of 20 μL. qPCR cycling conditions were: initial denaturation at 95 °C for 3 min; 35 cycles: denaturation 95 °C for 30 s, primer annealing at 59 °C for 30 s, and elongation at 72 °C for 270 s.

The primers were designed previously. The amount of mtDNA damage was calculated per 10,000 bp according to Equation:

mtDNA damage=1 − (2^−(Δlong−Δshort)^) × 10,000/fragment length (1)

where Δ long = Cq control − Cq experiment for the long fragment and Δ short = Cq control − cq experiment for the short fragment.

Primers for long mtDNA fragments were selected using the BLAST program.

The primer sequences were as follows (Table 4):

### 4.7. Assessing the Bacterial Composition of the Gut Microbiome

To analyze the microbiome of mice, we performed targeted sequencing of the 16S rRNA gene on the Ion Torrent PGM platform. For the most complete subsequent phylogenetic analysis, the sequence of the V3 hypervariable region of the 16S rRNA gene was chosen, which we limited to the universal primers 337F and 518R. Targeted amplification was performed using the 5× ScreenMix-HS Master Mix kit (Evrogen, Moscow, Russia) in the following regime: 94 °C for 4 min; 37 cycles of 94 °C for 30 s; 53 °C for 30 s; and 72 °C for 30 s; and final elongation at 72 °C for 5 min.

After PCR, amplicons were purified on AMPureXP magnetic beads (Beckman Coulter, Brea, CA, USA). The NEB-Next Fast DNA Library Prep Kit (New England Biolabs, Ipswich, MA, USA) was used to prepare the libraries following the sample preparation protocol. After barcodes were attached to the libraries and their quality was checked, we proceeded to the stage of emulsion PCR on the OneTouch 2 system (Thermo Fisher Scientific, Madison, WI, USA). Sequencing was performed with the Ion PGM Hi-Q View Sequencing Kit (Thermo Fisher Scientific, Madison, WI, USA) using the Ion Torrent PGM system.

The resulting sequences were converted from BAM format to FASTQ format for each read. Bioinformatics data analysis was performed using the R programming language in the R-studio environment. All read preprocessing, such as demultiplexing, filtering, and quality control was performed using VSEARCH v.2.8.2 software. The search for operational taxonomic units of the OTU and the creation of the OTU table were performed using the UNOISE2 algorithm. Reads were filtered using the maximum expected error cutoff of 1.0. Species-level taxonomy was assigned using 100% identity with amplicon sequence variants using the SILVA database version 132 (https://www.arb-silva.de, accessed on 26 December 2021). Taxonomic identification was carried out using the DADA2 package. The DADA2 package provides a built-in implementation of the Naive Bayes classifier method for this purpose.

### 4.8. Statistical Analysis

Statistical analysis was performed using Statistica 10 (StatSoft. Inc., Tulsa, OK, USA). The results were expressed as means ± SEM. The results of the physiological tests were analyzed by nonparametric U test. For calculation of normalized expression and copy number of mtDNA, standard Bio-Rad CFX Manager 3.1 (Bio-Rad Laboratories, Hercules, CA, USA) software was used.

## 5. Conclusions

In the course of our study, we showed the pathogenic effect of cisplatin on the cells of the nervous system. The inhibitory effect of cisplatin was demonstrated on the deterioration of the cognitive abilities of experimental animals, which was found in the course of physiological tests. Also, cisplatin reduced the level of expression of genes of the antioxidant system in the hippocampus and cortex. We have shown that cisplatin induces mitophagy due to mtDNA damage. Cisplatin also influenced the composition of the intestinal microbiome. We also showed the neuroprotective effect of MB and AzB; they prevented the destructive effects caused by cisplatin, the level of antioxidant system genes in the cortex and hippocampus, and also normalized the composition of the intestinal microbiome of mice. During our study, we noticed that AzB did not affect cognitive parameters, unlike MB, which improved the cognitive abilities of animals. In this regard, MB and AzB are interesting for careful study as drugs that can help people who have undergone chemotherapy to restore their level of cognitive functions, as well as drugs that prevent the deterioration of brain cells.

## Figures and Tables

**Table 1:** Results of the physiological test “Open field”.

Group Name	Hole-Poking	Grooming (Time)	Grooming (Number)	Center (Time)	Center (Number)	Rearing	Defecation	Horizontal Activity
Control	1 ± 0.7	15 ± 6.4	4.25 ± 0.9	14 ± 8.4	5.25 ± 2.9	21 ± 2.9	1.5 ± 0.7	104.5 ± 30.5
Saline injections	2.1 ± 0.7	28.4 ± 0.4	8.25 ± 0.9	7.9 ± 1.7	3.1 ± 0.5	20.75 ± 6.4	1 ± 0.3	73.6 ± 16.8
Cisplatin injections	2.2 ± 0.8	17.9 ± 5.4	6.3 ± 1.3	2.3 ± 1.1	1.3 ± 0.6	6.8 ± 1.6	0.5 ± 0.4	50.3 ± 14.9
Cisplatin + MB	2.5 ± 0.6	77.3 ± 30.1	7.5 ± 1.1	4.1 ± 2.3	1.5 ± 0.8	8.5 ± 2.7	0.4 ± 0.2	63.1 ± 9.8
Cisplatin + AzB	1.5 ± 0.4	15 ± 2.1 **	5.4 ± 0.5	4.75 ± 1.2	1.75 ± 0.4	7.75 ± 1.6	0.4 ± 0.4	54.1 ± 9.3

** *p* < 0.01 groups were compared using the U test. Differences are statistically significant between the Cisplatin + MB and Cisplatin + AzB groups.

**Table 2:** Scheme of Morris water maze experiment for study of reference memory with indication of starting point and goal platform.

Acquisition. Start Located in the SW				
Day	Trial 1	Trial 2	Trial 3	Trial 4
1	N	E	SE	NW
2	SE	N	NW	E
3	NW	SE	E	N
4	E	NW	N	SE
5	N	SE	E	NW
6 (Probe)	NE	-	-	-
Reversal. Start located in the NE				
Day	Trial 1	Trial 2	Trial 3	Trial 4
1	S	W	NW	SE
2	NW	S	SE	W
3	SE	NW	W	S
4	W	SE	S	NW
5	S	NW	W	SE
6 (Probe)	SW	-	-	-

**Table 3:** Primers that were used to analyze gene expression.

Gene	Accession Number	Primers Sequence (5′–3′)
*Nfe2l2*	NM_010902.4	Forward = CTCTCTGAACTCCTGGACGG Reserve = GGGTCTCCGTAAATGGAAG
*Bdnf*	NM_007540.4	Forward = AAGGACGCGGACTTGTACAC Reserve = CGCTAATACTGTCACACACGC
*Mtor*	NM_020009.2	Forward = AGATAAGCTCACTGGTCGGG Reserve = GTGGTTTTCCAGGCCTCAGT
*Akt1*	NM_009652.3	Forward = TGATCAAGATGACAGCATGGAGTG Reserve = GATGATCCATGCGGGGCTT
*Gclc*	NM_010295.2	Forward = GCAGCTTTGGGTCGCAAGTAG Reserve = TGGGTCTCTTCCCAGCTCAGT
*Gpx*	NM_008160.6	Forward = AGTCCACCGTGTATGCCTTCT Reserve = GAGACGCGACATTCTCAATGA
*Sod2*	NM_013671.3	Forward = CAGACCTGCCTTACGACTATGG Reserve = CTCGGTGGCGTTGAGATTGTT
*P62*	U17961.1	Forward = GCCAGAGGAACAGATGGAGT Reserve = TCCGATTCTGGCATCTGTAG
*Prdx3*	NM_007452.2	Forward = TGGCTTGATCGTAGGGGACT Reserve = GTGGTTTGGGCCACATGAAC
*Txnr2*	NM_013711.3	Forward = GATCCGGTGGCCTAGCTTG Reserve = TCGGGGAGAAGGTTCCACAT
*Pink1*	NM_026880.2	Forward = GAGCAGACTCCCAGTTCTCG Reserve = GTCCCACTCCACAAGGATGT
*Gapdh* (reference)	NM_001289726.1	Forward = GGCTCCCTAGGCCCCTCCTG Reserve = TCCCAACTCGGCCCCCAACA
*18s* (reference)	NR_003278.3	Forward = CGGCTACCACATCCAAGGAA Reserve = GCTGGAATTACTGTGGCT

**Table 4:** Primers that were used to analyse measurement of mtDNA damage (Accession number for mtDNA *Mus musculus* NC_005089.1).

mtDNAFragments	Primers Sequence (5′–3′)
1 long	Forward = TAAATTTCGTGCCAGCCACC Reserve = ATGCTACCTTTGCACGGTCA
2 long	Forward = ACGAGGGTCCAACTGTCTCTTA Reserve = CCGGCTGCGTATTCTACGTT
3 long	Forward = CTAGCAGAAACAAACCGGGC Reserve = TTAGGGCTTTGAAGGCTCGC
4 long	Forward = TCATTCTTCTACTATCCCCAATCC Reserve = TGGTTTGGGAGATTGGTTGATG
5 long	Forward = CCCCAATCCCTCCTTCCAAC Reserve = GGTGGGGAGTAGCTCCTTCTT
6 long	Forward = AAGAAGGAGCTACTCCCCACC Reserve = GTTGACACGTTTTACGCCGA
short (reference)	Forward = ACGAGGGTCCAACTGTCTCTTA Reserve = AGCTCCATAGGGTCTTCTCGT

## Data Availability

Data is contained within the article.

## References

[1] Cherdyntseva N.V., Kzhishkovskaya Y.G., Stakheeva M.N., Litvyakov N.V., Savelyeva O.E., Mitrofanova I.V., Stepanov I.V., Grachev A.N., Gerashchenko T.S., Zavyalova M.V. (2015). The Immune System and the Effectiveness of Antitumor Treatment. http://vital.lib.tsu.ru/vital/access/manager/Repository/vtls:000522451.

[2] Zhang Y. (2018). Cell toxicity mechanism and biomarker. Clin. Transl. Med..

[3] Dasari S., Tchounwou P.B. (2014). Cisplatin in cancer therapy: Molecular mechanisms of action. Eur. J. Pharmacol..

[4] Rosenberg B.H., Vancamp L., Trosko J.E., Mansour V.H. (1969). Platinum Compounds: A New Class of Potent Antitumour Agents. Nature.

[5] Gelasco A., Lippard S.J. (1998). NMR solution structure of a DNA dodecamer duplex containing a cis-diammineplatinum(II) d(GpG) intrastrand cross-link, the major adduct of the anticancer drug cisplatin. Biochemistry.

[6] Galea A.M., Murray V. (2002). The interaction of cisplatin and analogues with DNA in reconstituted chromatin. Biochim. Biophys. Acta BBA-Gene Struct. Expr..

[7] Guimaraes C.A., Linden R. (2004). Programmed cell deaths. Apoptosis and alternative deathstyles. J. Biol. Inorg. Chem..

[8] Kole A.J., Knight E.R., Deshmukh M. (2011). Activation of Apoptosis by Cytoplasmic Microinjection of Cytochrome *c*. J. Vis. Exp..

[9] Lomeli N., Di K., Czerniawski J., Guzowski J.F., Bota D.A. (2017). Cisplatin-induced mitochondrial dysfunction is associated with impaired cognitive function in rats. Free Radic. Biol. Med..

[10] Qi L., Luo Q., Zhang Y., Jia F., Zhao Y., Wang F. (2019). Advances in Toxicological Research of the Anticancer Drug Cisplatin. Chem. Res. Toxicol..

[11] Santos N.A.G.D., Ferreira R.S., Santos A.C.D. (2020). Overview of cisplatin-induced neurotoxicity and ototoxicity, and the protective agents. Food Chem. Toxicol..

[12] Kholodova N.B., Sotnikov V.M., Dobrovol’Skaia N.I., Ponkratova I.A. (2014). Aspects of encephalopathy in oncologic patients after chemotherapy. Zhurnal Nevrol. I PsikhiatriiIm. SS Korsakova.

[13] Ongnok B., Chattipakorn N., Chattipakorn S.C. (2020). Doxorubicin and cisplatin induced cognitive impairment: The possible mechanisms and interventions. Exp. Neurol..

[14] Akman T., Akman L., Erbaş O., Terek M.C., Taskiran D., Ozsaran A. (2015). The Preventive Effect of Oxytocin to Cisplatin-Induced Neurotoxicity: An Experimental Rat Model. BioMed Res. Int..

[15] Vindya N.S., Mohamad A., Razdan R. (2019). Allantoin attenuates deficits of behavioural and motor nerve conduction in an animal model of cisplatin-induced neurotoxicity in rats. Anim. Model Exp. Med..

[16] Almutairi M.M., Alanazi W., Alshammari M.A., Alotaibi M.R., Alhoshani A.R., Al-Rejaie S.S., Hafez M.M., Al-Shabanah O.A. (2017). Neuro-protective effect of rutin against Cisplatin-induced neurotoxic rat model. BMC Complement. Altern. Med..

[17] Sharawy N., Rashed L., Youakim M.F. (2015). Evaluation of multi-neuroprotective effects of erythropoietin using cisplatin induced peripheral neurotoxicity model. Exp. Toxicol. Pathol..

[18] Gong S., Feng Y., Zeng Y., Zhang H., Pan M., He F., Wu R., Chen J., Lu J., Zhang S. (2021). Gut microbiota accelerates cisplatin-induced acute liver injury associated with robust inflammation and oxidative stress in mice. J. Transl. Med..

[19] Chambers L.M., Esakov E., Braley C., Sangwan N., Vargas R., Rose P., Lathia J., Michener C., Reizes O. (2020). Cisplatin chemotherapy impacts the gut microbiome in a preclinical murine model of epithelial ovarian cancer. Gynecol. Oncol..

[20] Wu C.-H., Ko J.-L., Liao J.-M., Huang S.-S., Lin M.-Y., Lee L.-H., Chang L.-Y., Ou C.-C. (2019). D-methionine alleviates cisplatin-induced mucositis by restoring the gut microbiota structure and improving intestinal inflammation. Ther. Adv. Med. Oncol..

[21] Morais L.H., Schreiber H.L., Mazmanian S.K. (2021). The gut microbiota-brain axis in behaviourand brain disorders. Nat. Rev. Microbiol..

[22] Rebillard A., Rioux-Leclercq N., Muller C., Bellaud P., Jouan F., Meurette O., Jouan E., Vernhet L., Le Quément C., Carpinteiro A. (2008). Acid sphingomyelinase deficiency protects from cisplatin-induced gastrointestinal damage. Oncogene.

[23] Patankar J.V., Becker C. (2020). Cell death in the gut epithelium and implications for chronic inflammation. Nat. Rev. Gastroenterol. Hepatol..

[24] Solas M., I Milagro F., Ramírez M.J., Martínez J.A. (2017). Inflammation and gut-brain axis link obesity to cognitive dysfunction: Plausible pharmacological interventions. Curr. Opin. Pharmacol..

[25] Tucker D., Lu Y., Zhang Q. (2017). From Mitochondrial Function to Neuroprotection—an Emerging Role for Methylene Blue. Mol. Neurobiol..

[26] Gureev A.P., Syromyatnikov M.Y., Ignatyeva D.A., Valuyskikh V.V., Solodskikh S.A., Panevina A.V., Gryaznova M.V., Kokina A.V., Popov V.N. (2020). Effect of long-term methylene blue treatment on the composition of mouse gut microbiome and its relationship with the cognitive abilities of mice. PLoS ONE.

[27] Sadovnikova I.S., Gureev A.P., Ignatyeva D.A., Gryaznova M.V., Chernyshova E.V., Krutskikh E.P., Novikova A.G., Popov V.N. (2021). Nrf2/ARE Activators Improve Memory in Aged Mice via Maintaining of Mitochondrial Quality Control of Brain and the Modulation of Gut Microbiome. Pharmaceuticals.

[28] Sekeres M.J., Winocur G., Moscovitch M. (2018). The hippocampus and related neocortical structures in memory transformation. Neurosci. Lett..

[29] Cowan N. (2008). What are the differences between long-term, short-term, and working memory?. Prog. Brain Res..

[30] Wood A.M., Linley P.A., Maltby J., Kashdan T.B., Hurling R. (2011). Using personal and psychological strengths leads to increases in well-being over time: A longitudinal study and the development of the strengths use questionnaire. Personal. Individ. Differ..

[31] Bliss T.V.P., Lømo T. (1973). Long-lasting potentiation of synaptic transmission in the dentate area of the anaesthetized rabbit following stimulation of the perforant path. J. Physiol..

[32] Gureev A.P., Popov V.N., Starkov A.A. (2020). Crosstalk between the mTOR and Nrf2/ARE signaling pathways as a target in the improvement of long-term potentiation. Exp. Neurol..

[33] Zakria M., Ahmad N., Al Kury L.T., Alattar A., Uddin Z., Siraj S., Ullah S., Alshaman R., Khan M.I., Shah F.A. (2021). Melatonin rescues the mice brain against cisplatin-induced neurodegeneration, an insight into antioxidant and anti-inflammatory effects. NeuroToxicology.

[34] Shabani M., Larizadeh M.H., Parsania S., Hajali V., Shojaei A. (2012). Evaluation of Destructive Effects of Exposure to Cisplatin During Developmental Stage: No Profound Evidence for Sex Differences in Impaired Motor and Memory Performance. Int. J. Neurosci..

[35] Chen C., Zhang H., Xu H., Zheng Y., Wu T., Lian Y. (2019). Ginsenoside Rb1 ameliorates cisplatin-induced learning and memory impairments. J. Ginseng. Res..

[36] Oz M., Atalik K.E.N., Yerlikaya F.H., Demir E.A. (2015). Curcumin alleviates cisplatin-induced learning and memory impairments. Neurobiol. Learn. Mem..

[37] Kandeil M.A., Gomaa S.B., Mahmoud M.O. (2020). The effect of some natural antioxidants against cisplatin-induced neurotoxicity in rats: Behavioral testing. Heliyon.

[38] Shabani M., Nazeri M., Parsania S., Razavinasab M., Zangiabadi N., Esmaeilpour K., Abareghi F. (2012). Walnut consumption protects rats against cisplatin-induced neurotoxicity. NeuroToxicology.

[39] Hosseinzadeh M., Alizadeh A., Heydari P., Kafami M., Hosseini M., Beheshti F., Marefati N., Ghanbarabadi M. (2020). Effect of vitamin E on cisplatin-induced memory impairment in male rats. Acta Neuropsychiatr..

[40] Hussien M., Yousef M.I. (2021). Impact of ginseng on neurotoxicity induced by cisplatin in rats. Environ. Sci. Pollut. Res. Int..

[41] Ma J., Huo X., Jarpe M.B., Kavelaars A., Heijnen C.J. (2018). Pharmacological inhibition of HDAC6 reverses cognitive impairment and tau pathology as a result of cisplatin treatment. Acta Neuropathol. Commun..

[42] Chiu G.S., Boukelmoune N., Chiang A.C., Peng B., Rao V., Kingsley C., Liu H.-L., Kavelaars A., Kesler S.R., Heijnen C.J. (2018). Nasal administration of mesenchymal stem cells restores cisplatin-induced cognitive impairment and brain damage in mice. Oncotarget.

[43] Wahdan S.A., Elsherbiny D.A., Azab S.S., El-Demerdash E. (2021). Piceatannol ameliorates behavioural, biochemical and histological aspects in cisplatin-induced peripheral neuropathy in rats. Basic Clin. Pharmacol. Toxicol..

[44] Chiu G.S., Maj M.A., Rizvi S., Dantzer R., Vichaya E.G., Laumet G., Kavelaars A., Heijnen C.J. (2017). Pifithrin-µ Prevents Cisplatin-Induced Chemobrain by Preserving Neuronal Mitochondrial Function. Cancer Res..

[45] El-Deeb O.S., Soliman G.M., Elesawy R.O. (2020). Linagliptin, the dipeptidyl peptidase-4 enzyme inhibitor, lessens CHOP and GRP78 biomarkers levels in cisplatin-induced neurobehavioral deficits: A possible restorative gateway. J. Biochem. Mol. Toxicol..

[46] Salih N.A., Al-Baggou B.K. (2020). Effect of memantine hydrochloride on cisplatin-induced neurobehavioral toxicity in mice. Acta Neurol. Belg..

[47] Borbélyová V., Renczés E., Chovanec M., Mego M., Celec P. (2020). Transient effects of chemotherapy for testicular cancer on mouse behaviour. Sci. Rep..

[48] John T., Lomeli N., Bota D.A. (2017). Systemic cisplatin exposure during infancy and adolescence causes impaired cognitive function in adulthood. Behav. Brain Res..

[49] Cankara F.N., Günaydın C., Çelik Z.B., Şahin Y., Pekgöz Ş., Erzurumlu Y., Gülle K. (2021). Agomelatine confers neuroprotection against cisplatin-induced hippocampal neurotoxicity. Metab. Brain Dis..

[50] Bazer D., Kowalska A. (2020). NCMP-15. CNS lymphoma: The great mimicker. NeuroOncology.

[51] Jangra A., Kwatra M., Singh T., Pant R., Kushwah P., Ahmed S., Dwivedi D., Saroha B., Lahkar M. (2016). Edaravone alleviates cisplatin-induced neurobehavioral deficits via modulation of oxidative stress and inflammatory mediators in the rat hippocampus. Eur. J. Pharmacol..

[52] Troy L., McFarland K., Littman-Power S., Kelly B.J., Walpole E.T., Wyld D., Thomson D. (2000). Cisplatin-based therapy: A neurological and neuropsychological review. Psychooncology.

[53] Yi L.-T., Dong S.-Q., Wang S.-S., Chen M., Li C.-F., Geng D., Zhu J.-X., Liu Q., Cheng J. (2019). Curcumin attenuates cognitive impairment by enhancing autophagy in chemotherapy. Neurobiol. Dis..

[54] Amidi A., Hosseini S.M.H., Leemans A., Kesler S.R., Agerbaek M., Wu L., Zachariae R. (2017). Changes in Brain Structural Networks and Cognitive Functions in Testicular Cancer Patients Receiving Cisplatin-Based Chemotherapy. J. Natl. Cancer Inst..

[55] Andres A.L., Gong X., Di K., Bota D.A. (2014). Low-doses of cisplatin injure hippocampal synapses: A mechanism for ‘chemo’ brain?. Exp. Neurol..

[56] Tang A.H., Neufeld T.P., Rubin G.M., A Müller H. (2001). Transcriptional regulation of cytoskeletal functions and segmentation by a novel maternal pair-rule gene, lilliputian. Development.

[57] Ersahin T., Tuncbag N., Cetin-Atalay R. (2015). The PI3K/AKT/mTOR interactive pathway. Mol. BioSyst..

[58] Schratt G.M., Nigh E.A., Chen W.G., Hu L., Greenberg M.E. (2004). BDNF Regulates the Translation of a Select Group of mRNAs by a Mammalian Target of Rapamycin-Phosphatidylinositol 3-Kinase-Dependent Pathway during Neuronal Development. J. Neurosci..

[59] Sun Y.-X., Yang J., Wang P.-Y., Li Y.-J., Xie S.-Y., Sun R.-P. (2013). Cisplatin regulates SH-SY5Y cell growth through downregulation of BDNF via miR-16. Oncol. Rep..

[60] Wang Y., Liu N., Lu B. (2019). Mechanisms and roles of mitophagy in neurodegenerative diseases. CNS Neurosci. Ther..

[61] Fivenson E.M., Lautrup S.H., Sun N., Scheibye-Knudsen M., Stevnsner T., Nilsen H., Bohr V.A., Fang E.F. (2017). Mitophagy in neurodegeneration and aging. Neurochem. Int..

[62] Husain K., Morris C., Whitworth C., Trammell G., Rybak L., Somani S. (1998). Protection by ebselen against cisplatin-induced nephrotoxicity: Antioxidant system. Mol. Cell. Biochem..

[63] Garibay-Valdez E., Cicala F., Martinez-Porchas M., Gómez-Reyes R., Vargas-Albores F., Gollas-Galván T., Martínez-Córdova L.R., Calderón K. (2021). Longitudinal variations in the gastrointestinal microbiome of the white shrimp. Litopenaeusvannamei. PeerJ.

[64] Vogt N.M., Kerby R.L., Dill-McFarland K.A., Harding S.J., Merluzzi A.P., Johnson S.C., Carlsson C.M., Asthana S., Zetterberg H., Blennow K. (2017). Gut microbiome alterations in Alzheimer’s disease. Sci. Rep..

[65] Iljazovic A., Roy U., Gálvez E.J., Lesker T.R., Zhao B., Gronow A., Amend L., Will S.E., Hofmann J.D., Pils M.C. (2021). Perturbation of the gut microbiome by *Prevotella* spp. enhances host susceptibility to mucosal inflammation. Mucosal Immunol..

[66] Larsen J.M. (2017). The immune response to Prevotella bacteria in chronic inflammatory disease. Immunology.

[67] Li Y.-F., Gong X.-L., Chen S.-X., Wang K., Jiang Y.-H. (2021). Deviations in the gut microbiota of neonates affected by maternal group B Streptococcus colonization. BMC Microbiol..

[68] Barandouzi Z.A., Starkweather A.R., Henderson W., Gyamfi A., Cong X.S. (2020). Altered Composition of Gut Microbiota in Depression: A Systematic Review. Front. Psychiatry.

[69] Schirmer R.H., Adler H., Pickhardt M., Mandelkow E. (2011). Lest we forget you–methylene blue. Neurobiol. Aging.

[70] Martinez J.L., Jensen R.A., Vasquez B.J., McGuinness T., McGaugh J.L. (1978). Methylene blue alters retention of inhibitory avoidance responses. Physiol. Psychol..

[71] Callaway N.L., Riha P.D., Wrubel K.M., McCollum D., Gonzalez-Lima F. (2002). Methylene blue restores spatial memory retention impaired by an inhibitor of cytochrome oxidase in rats. Neurosci. Lett..

[72] Rodriguez P., Zhou W., Barrett D.W., Altmeyer W., Gutierrez J.E., Li J., Lancaster J.L., Gonzalez-Lima F., Duong T.Q. (2016). Multimodal Randomized Functional MR Imaging of the Effects of Methylene Blue in the Human Brain. Radiology.

[73] Gureev A.P., Shaforostova E.A., Popov V.N., Starkov A.A. (2019). Methylene blue does not bypass Complex III antimycin block in mouse brain mitochondria. FEBS Lett..

[74] Atamna H., Atamna W., Al-Eyd G., Shanower G., Dhahbi J.M. (2015). Combined activation of the energy and cellular-defense pathways may explain the potent anti-senescence activity of methylene blue. Redox Biol..

[75] Zhang M., An C., Gao Y., Leak R.K., Chen J., Zhang F. (2013). Emerging roles of Nrf2 and phase II antioxidant enzymes in neuroprotection. Prog. Neurobiol..

[76] Gureev A.P., Sadovnikova I.S., Starkova N.N., Starkov A.A., Popov V.N. (2020). p62-Nrf2-p62 Mitophagy Regulatory Loop as a Target for Preventive Therapy of Neurodegenerative Diseases. Brain Sci..

[77] Murata H., Takamatsu H., Liu S., Kataoka K., Huh N.-H., Sakaguchi M. (2015). NRF2 Regulates PINK1 Expression under Oxidative Stress Conditions. PLoS ONE.

[78] Sha C., Barrans S., Cucco F., Bentley M.A., Care M.A., Cummin T., Kennedy H., Thompson J.S., Uddin R., Worrillow L. (2019). Molecular High-Grade B-Cell Lymphoma: Defining a Poor-Risk Group That Requires Different Approaches to Therapy. J. Clin. Oncol..

[79] Bendavid I., Singer P., Theilla M., Themessl-Huber M., Sulz I., Mouhieddine M., Schuh C., Mora B., Hiesmayr M. (2017). NutritionDay ICU: A 7 year worldwide prevalence study of nutrition practice in intensive care. Clin. Nutr..

[80] Tufekci K.U., CiviBayin E., Genc S., Genc K. (2011). The Nrf2/ARE Pathway: A Promising Target to Counteract Mitochondrial Dysfunction in Parkinson’s Disease. Parkinson’s Dis..

[81] Warth A., Goeppert B., Bopp C., Schirmacher P., Flechtenmacher C., Burhenne J. (2009). Turquoise to dark green organs at autopsy. Virchows Arch..

[82] Petzer A., Harvey B.H., Wegener G., Petzer J.P. (2012). Azure B, a metabolite of methylene blue, is a high-potency, reversible inhibitor of monoamine oxidase. Toxicol. Appl. Pharmacol..

[83] Biberoglu K., Yuksel M., Tacal O. (2018). Azure B affects amyloid precursor protein metabolism in PS70 cells. Chem. Interact..

[84] Pakavathkumar P., Sharma G., Kaushal V., Foveau B., Leblanc A.C. (2015). Methylene Blue Inhibits Caspases by Oxidation of the Catalytic Cysteine. Sci. Rep..

[85] Čulo F., Sabolović D., Somogyi L., Marušić M., Berbiguier N., Galey L. (1991). Anti-tumoral and anti-inflammatory effects of biological stains. Agents Actions.

[86] Haouzi P., McCann M., Tubbs N. (2020). Azure B as a novel cyanide antidote: Preclinical in-vivo studies. Toxicol. Rep..

[87] Kalueff A.V., Tuohimaa P. (2005). Mouse grooming microstructure is a reliable anxiety marker bidirectionally sensitive to GABAergic drugs. Eur. J. Pharmacol..

